# Early Recurrence of Mediastinal Germ Cell Tumor With Pathological Complete Response to Chemotherapy: A Case Report

**DOI:** 10.1155/crpe/6687345

**Published:** 2026-04-01

**Authors:** Risa Kanai, Satoshi Yokoyama, Daisuke Fukao, Masahiko Sakabe

**Affiliations:** ^1^ Department of Pediatric Surgery, Japanese Red Cross Society Wakayama Medical Center, 4-20 Komatsubara-dori, Wakayama-shi, Wakayama, 640-8558, Japan; ^2^ Department of Pediatrics, Japanese Red Cross Society Wakayama Medical Center, 4-20 Komatsubara-dori, Wakayama-shi, Wakayama, 640-8558, Japan

## Abstract

Primary malignant mediastinal germ cell tumors (GCTs) are rare pediatric tumors that have a poorer prognosis than GCTs occurring elsewhere in the body. Due to their rarity and aggressiveness, they are often locally advanced and unresectable at diagnosis, and currently, there is no standard optimal treatment approach. Herein, we present a rare case of pediatric anterior mediastinal yolk sac tumor (YST) diagnosed as pathological complete response (CR) to chemotherapy but prematurely required pulmonary metastasectomy of mixed GCT consisting of seminoma and YST. Testicular GCTs can relapse as tumors of subtypes that are pathologically different from the primary tumor, each having distinct pathogenesis, treatment modality, and prognosis; however, this phenomenon is seldom reported in mediastinal GCTs. This report reviewed the discordance of histological composition between primary and metastatic tumors. When treating mediastinal nonseminomatous GCTs, the possibility of early recurrence should be considered, even if the primary tumor is completely resected and diagnosed as pathological CR to chemotherapy.

## 1. Introduction

Germ cell tumors (GCTs) comprise 4%–10% of all mediastinal tumors among adults and up to 25% among children [1]. Only 1%–5% of all GCTs arise in extragonadal locations, predominantly in the mediastinum [1, 2]. Mediastinal nonseminomatous GCTs have a 5‐year overall survival rate of only 40%–50%, placing them in the International Germ Cell Cancer Collaborative Group (IGCCCG) poor‐prognosis group [2, 3]. Herein, we present a rare case of pediatric anterior mediastinal yolk sac tumor (YST) diagnosed as pathological complete response (CR) to chemotherapy but prematurely required pulmonary metastasectomy of mixed GCT consisting of seminoma and YST.

## 2. Case Presentation

**FIGURE 1:**
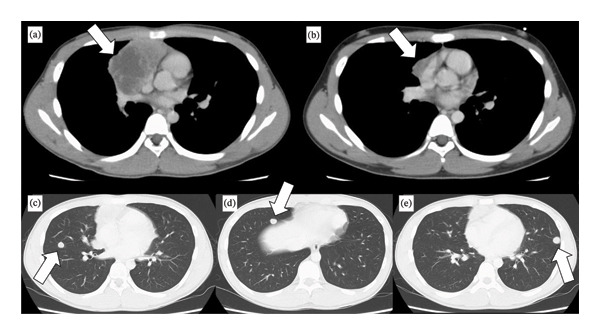
Computed tomography (CT) revealed a mass in the right anterior mediastinum and multiple bilateral lung nodules: (a) before chemotherapy, (b) at surgery after chemotherapy, and (c–e) three representative CT images of the pulmonary metastases.

**FIGURE 2:**
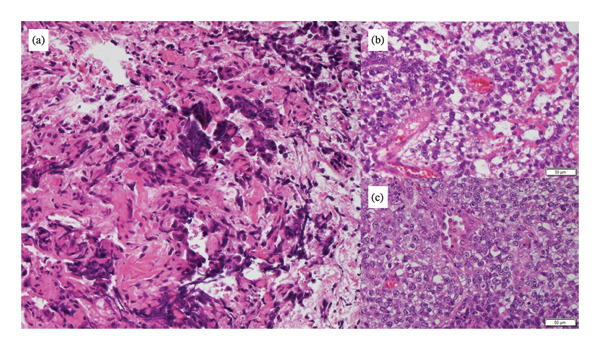
Pathological findings (hematoxylin and eosin staining, 20x). (a) The primary mediastinal tumor was diagnosed as YST at the initial diagnosis. Immunostaining of alpha‐fetoprotein. (b‐c) The metastatic pulmonary tumor was diagnosed as mixed GCT. (b) YST components and (c) seminoma components.

A 13‐year‐old boy was brought to our hospital with a history of cough and chest pain lasting for 1 month. He was diagnosed as having a mass on the chest X‐ray examination. Computed tomography (CT) scan revealed a large mass in the right anterior mediastinum (Figure 1(a)) and multiple bilateral lung nodules (Figures 1(c), 1(d), and 1(e)). The mediastinal tumor infiltrated into the ascending aorta, superior vena cava, right superior pulmonary vein, and right upper lobe. Seven pulmonary nodules were identified, three in the right lung and four in the left lung. We performed a comprehensive systemic work‐up for diagnosis and staging. Serum tumor markers demonstrated significantly high levels of α‐fetoprotein (AFP) (3603 ng/mL) and normal β‐human chorionic gonadotropin (β‐hCG). Regarding the testes, physical examination, ultrasonography, and CT scan revealed no abnormalities, and no tumorous lesions were observed in the retroperitoneum. These findings support the diagnosis of a primary mediastinal tumor with multiple pulmonary metastases. Thoracoscopic biopsy of the mediastinal tumor revealed a diagnosis of YST (Figure 2(a)). Our multidisciplinary team planned initial chemotherapy with carboplatin, etoposide, and bleomycin (JEB) and considered surgical indications during chemotherapy. After four courses of chemotherapy, CT scan revealed that the mediastinal tumor had significantly reduced in size (Figure 1(b)), all multiple pulmonary metastases disappeared, and serum tumor markers normalized.

**FIGURE 3:**
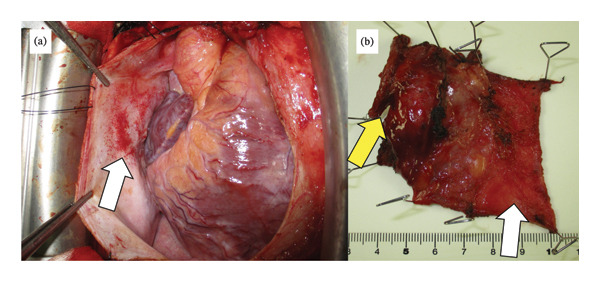
Intraoperative findings. (a) The tumor invaded the pericardium, except for intrapericardial invasion. (b) The specimen of the mediastinal tumor included the partial right upper lobe (yellow arrow) and the partial pericardium (white arrow).

**FIGURE 4:**
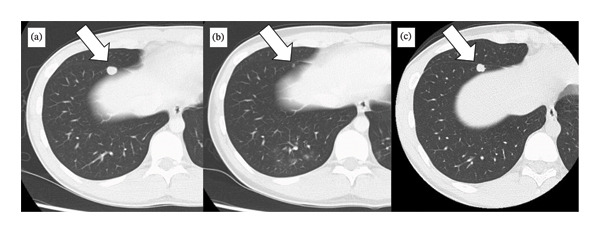
A pulmonary metastasis in the right lower lobe: (a) before chemotherapy, (b) at surgery of the mediastinal tumor, and (c) at metastasectomy after additional chemotherapy.

Subsequently, excision of the residual mediastinal tumor was performed via median sternotomy with cardiopulmonary bypass on standby. Although significant fibrosis complicated tissue dissection, intrapericardial invasion was absent, and right superior pulmonary vein and right phrenic nerve could be preserved (Figure 3(a)). Complete tumor resection was successfully performed, including pulmonary wedge resection and partial pericardiotomy, followed by patch repair (Figure 3(b)). There were no obvious pulmonary metastases in the right lobes. Histopathology demonstrated fibrinoid degeneration and necrosis, which was diagnosed as pathological CR to chemotherapy. After resection, serum tumor markers remained within the normal range. Despite an additional two courses of chemotherapy, a subsequent CT scan at 3 months postsurgery revealed a regrowth of one nodule in the right lower lobe (Figures 4(a), 4(b), and 4(c)). No other apparent tumors were detected in other lobes or systemic organs, and no re‐elevation of tumor markers was observed at the time. Pulmonary wedge resection was performed, and histopathology revealed viable tumors consisting of 90% seminoma and 10% YST, confirming mixed GCT (Figures 2(b) and 2(c)). Thereafter, he was restarted on chemotherapy to maintain remission.

## 3. Discussion

The IGCCCG classifies patients with GCTs into good‐, intermediate‐, and poor‐risk categories. Within the poor‐risk group, young adults with mediastinal primary tumors have been reported to have particularly poor outcomes [1, 4]. Nonseminomatous mediastinal tumors, in particular, have a worse prognosis than seminomatous mediastinal tumors. Furthermore, among nonseminomatous tumors, mediastinal primary tumors also have a poorer prognosis than retroperitoneal primary tumors [3].

Due to the rarity and aggressiveness of mediastinal nonseminomatous GCTs, they are often locally advanced and unresectable at diagnosis, and there is not yet a standard optimal treatment approach. A widely employed multimodal strategy involves neoadjuvant chemotherapy followed by consolidation surgery. Tumor infiltration into adjacent respiratory and cardiovascular structures is common, and surgery typically requires extensive resection during cardiopulmonary bypass [1]. Overall survival improvement is strongly associated with complete tumor resection [5]; the resection achieves the removal of tumor tissue resistant to chemotherapy and provides a sample for histological examination, which facilitates the assessment of pathological response to chemotherapy and planning of further management.

Determining the optimal timing for tumor resection is essential. In patients with mediastinal nonseminomatous GCTs and distant metastases, chemotherapy is generally recommended if symptoms related to the primary lesion are clinically tolerable [5]. Our patient presented with multiple bilateral pulmonary metastases at initial diagnosis, prompting the initiation of chemotherapy. Consolidation surgery is typically planned after chemotherapy, taking into consideration tumor marker levels, tumor size reduction, the relationship with adjacent structures, and the resolution of metastatic lesions [1, 5]. In our case, surgery was considered following the resolution of all pulmonary metastases and normalization of tumor markers. A decrease in tumor markers, in particular, has been reported to correlate strongly with prognosis; specifically for mediastinal YSTs after chemotherapy, complete normalization of serum AFP levels occurs in fewer than 5% of the patients but remains an important prognostic factor [1].

GCTs are classified into tumors of one histological type (pure forms) and more than one histological type (mixed forms). The subtypes of GCTs encompass seminoma/germinoma, YST, immature teratoma, embryonal carcinoma, choriocarcinoma, and mixed GCT [6]. In our case, although the primary mediastinal tumor was diagnosed as YST, the metastatic pulmonary tumor turned out to be mixed GCT (seminoma and YST). Originally, at the initial diagnosis, the thoracoscopic biopsy evaluated only a portion of the mediastinal tumor, and it might have been mixed GCT, including YST. However, this result could not be confirmed because the mediastinal tumor had no viable tissue at the time of tumor resection after chemotherapy. We have analyzed the discrepancy in histology between the primary YST and the metastatic mixed GCT, in the context of the existing literature, and explored possible underlying mechanisms [6–10]. Among testicular GCTs, there is frequent discordance of the histological composition between primary and metastatic tumors; however, this is seldom reported in mediastinal GCTs [6]. Although multiple theories have been proposed to explain the cause of this phenomenon, it is most likely due to the common embryonic origin of all GCTs from primitive pluripotent germ cells, which can transform into different malignant cell types [7]. Because totipotent germ cells are the precursors of all GCTs, their forward and backward differentiation can result in histopathological differences between the primary and metastatic cancer cells [8]. This discordance may be caused by the inherent resistance of some GCT components to chemotherapy, resulting in their survival and selective proliferation [8, 9]. A similar condition in this respect, growing teratoma syndrome presents with enlarging teratoma masses with normalized tumor markers, occurring during or after systemic chemotherapy for the GCT treatment [10]. Our patient’s concomitant mediastinal YST and metastatic mixed GCT, which primarily consisted of seminoma, supports the theory of backward differentiation of YST into seminoma, which subsequently metastasized to the lung.

Moreover, the optimal management of patients with metastatic GCTs who achieve CR after first‐line chemotherapy remains debatable. Some retrospective cohort studies reported that most patients belonged to the good prognosis group in the IGCCCG classification. In these studies, among patients with metastatic GCTs who were treated with first‐line chemotherapy, 60% responded favorably (i.e., normalization of serum tumor marker levels with radiographic residuals) and up to 30% achieved a complete clinical response (i.e., normalization of serum tumor marker levels and residual masses < 1 cm) [4, 11]. For patients with testicular GCTs who achieve a complete clinical response, the current guidelines recommend ongoing surveillance based on the data from four retrospective cohort studies that reported recurrence in 41 out of 540 patients (8%) [12, 13]. Furthermore, most patients who relapse are salvageable with surgery and/or chemotherapy [4]. However, limited data are available for patients with mediastinal nonseminomatous GCTs that are classified as a poor prognosis group in the IGCCCG classification. Li Qin et al. demonstrated that patients who underwent complete resection of primary mediastinal GCTs experienced early recurrence within 6–11 months of resection (median: 8.0 months), highlighting the need for close follow‐up [14]. In our patient, serum tumor markers were normalized, and the pulmonary nodule was solitary, making it difficult to confirm recurrence. The nodule was, therefore, resected for diagnostic and therapeutic purposes, and histopathological examination confirmed recurrence. Currently, there is no standard established treatment approach for early‐recurrent metastatic mediastinal GCTs, and the relative benefits and risks of surgical resection versus second‐line chemotherapy remain unclear. The optimal strategy may depend on factors such as tumor burden, response to prior therapy, and timing of recurrence. Clinicians must stay vigilant regarding early recurrence, as prompt identification may impact subsequent treatment decisions. Further investigation is warranted, and a more extensive dataset should be accumulated.

In conclusion, mediastinal nonseminomatous GCTs in young adults are reported to have a poor prognosis and require close follow‐up. GCTs can relapse as tumors of subtypes that are pathologically different from the primary tumor, each having distinct pathogenesis, treatment modality, and prognosis. When treating these patients, the possibility of early recurrence should be considered, even if the primary tumor is completely resected and diagnosed as pathological CR to chemotherapy.

**Nomenclature**
GCT: Germ cell tumor
YST: Yolk sac tumor
CR: Complete response
IGCCCG: International Germ Cell Cancer Collaborative Group
CT: Computed tomography
AFP: α‐fetoprotein
β‐hCG: β‐human chorionic gonadotropin

## Author Contributions

Risa Kanai drafted the initial manuscript. Satoshi Yokoyama reviewed the manuscript and supervised the study. Daisuke Fukao and Masahiko Sakabe provided conceptual advice. Risa Kanai has overall responsibility for and guarantees the scientific integrity of this paper.

## Funding

No funding was received for this manuscript.

## Disclosure

All the authors gave final approval to the publication of the submitted version of this paper.

## Consent

The patient described in this case report gave written informed consent for the publication of this case report and the accompanying images.

## Conflicts of Interest

The authors declare no conflicts of interest.

## Data Availability

The data that support the findings of this study are available from the corresponding author upon reasonable request.
